# An Efficient Vector System to Modify Cells Genetically

**DOI:** 10.1371/journal.pone.0026380

**Published:** 2011-11-11

**Authors:** Huamin Han, Qingjun Liu, Wen He, Kristy Ong, Xiaoli Liu, Bin Gao

**Affiliations:** 1 CAS Key Laboratory of Pathogenic Microbiology and Immunology (CASPMI), Institute of Microbiology, Chinese Academy of Sciences, Beijing, People's Republic of China; 2 Graduate University of Chinese Academy of Sciences, Beijing, People's Republic of China; 3 UCL Institute of Child Health, London, United Kingdom; 4 Epigen Biotec Ltd, Beijing, People's Republic of China; 5 China-Japan Joint Laboratory of Molecular Immunology and Microbiology, Institute of Microbiology, Chinese Academy of Sciences, Beijing, People's Republic of China; Charité-Universitätsmedizin Berlin, Germany

## Abstract

The transfer of foreign genes into mammalian cells has been essential for understanding the functions of genes and mechanisms of genetic diseases, for the production of coding proteins and for gene therapy applications. Currently, the identification and selection of cells that have received transferred genetic material can be accomplished by methods, including drug selection, reporter enzyme detection and GFP imaging. These methods may confer antibiotic resistance, or be disruptive, or require special equipment. In this study, we labeled genetically modified cells with a cell surface biotinylation tag by co-transfecting cells with BirA, a biotin ligase. The modified cells can be quickly isolated for downstream applications using a simple streptavidin bead method. This system can also be used to screen cells expressing two sets of genes from separate vectors.

## Introduction

The transfer of foreign genes into eukaryotic cells, and in particular, into mammalian cells, plays an important role in our understanding of the function of coding genes and the regulatory components of non-coding sequences and also allows the production of coding proteins for therapeutic purposes and the development of strategies for gene therapy [1]. The rapid identification and selection of gene-modified cells are prerequisites for these applications.

Several systems have been developed for the selection of cells after gene transfer, including drug selection, GFP imaging and detection of other reporters [2], [3], [4], [5]. Antibiotic selection is the most commonly used method and is based on the growth advantage of the transfectants in the presence of a cytotoxic agent along with the death of the non-transduced cells. The widely-used antibiotic resistance genes include aminoglycoside phosphotransferase, dihydrofolate reductase (DHFR), hygromycin B phosphotransferase, puromycin-N-acetyl-transferase, blasticidin S deaminase, and glutamine synthetase (GS), which confer resistance to G418, methotrexate, hygromycin, puromycin, blasticidin, and methionine sulfoximine respectively [6], [7], [8], [9], [10], [11]. Isolation of gene-modified cells using this method requires several days to weeks and introduces undesirable drug resistance genes into the cells. Identification of transfected cells using reporter genes such as chloramphenicol acetyltransferase, alkaline phosphatase, β-galactosidase and firefly luciferase typically requires disruptive methods like cell permeabilization [7], [9], [12]. Green fluorescent protein (GFP) can be detected without cell permeabilization and is useful in fluorescence-activated cell sorting applications, but it can be toxic to cells.

Magnetic-activated cell sorting (MACS) is a simple solution for applications requiring the enrichment of cells of interest. MACS is dependent on the expression of a specific surface marker that can be recognized by a magnetic bead-tagged antibody. Gotoh et al. described eight streptavidin fusion genes as dominant selectable markers that can be combined with paramagnetic beads to select transfected cells [13]. Using MACS, it is possible to identify rare cell populations, separate large number of cells and large as many as 10^11^ cells in approximately 1 hour [14]. The immunomagnetic selection procedure is simple and rapid. This method yields a highly pure population of transfected cells and can be used for a wide range of biological applications. Several approaches have been put forwarded to develop simpler and faster selection strategies. Kawahara et al. have proposed a novel selection system called the antigen-mediated genetically modified cell amplification (AMEGA) system, which employs an antibody/receptor chimera that triggers a growth signal in response to a cognate antigen without antibiotic selection [15], [16], [17], [18], [19].

The association of Streptavidin with biotin is the strongest known non-covalent bond, which is several orders of magnitude stronger than that of antigen-antibody interactions. The biotin ligase, BirA, can catalyze the biotinylation of the ε-NH_2_ of a 13-amino acid peptide tag, a so-called minimal biotin acceptor sequence [20], [21] and has been widely used for biotinylation of a protein of interest. In this study, we take advantage of a biotin ligase enzyme to catalyze the biotinylation of a cell-surface peptide tag co-transferred into the same cell to create an efficient vector system for modifying cells genetically.

The selection system consists of two vectors; one contains a target gene and the biotin ligase BirA as a reporter, and the other contains a second target gene and a BirA substrate peptide linked to a truncated form of human low-affinity nerve growth factor receptor (**Δ**LNGFR). The target gene cassette in each vector is used to express the genes of interest. Once the lentivectors enter cells through transient transfection or infection, one or more of the target proteins are expressed. BirA is retained in the endoplasmic reticulum (ER) after expression, and it efficiently biotinylates the BirA tags fused to **Δ**LNGFR when they pass through the ER. The biotinylated **Δ**LNGFR is transported to the cell surface where it labels the target cell. The biotinylated cells have a high affinity for streptavidin and can be quickly and efficiently captured in vitro with streptavidin-labeled beads [22]. Using this method, highly pure populations of transfected cells can be obtained. Late-generation lentiviral vectors with advanced performance and safety profiles can efficiently transduce several types of nondividing and dividing cells. This method can be used to isolate mammalian cells modified by two sets of target genes from heterogeneous cell pools using magnetic streptavidin-labeled beads. This system has been used in multiple cell types, including the 293T, HCT-15, SupT1 and K42-91a, a mouse fibroblast cell line.

## Results

### 1. Cell surface expression of BirA enzyme substrate linked to a truncated LNGFR

**Figure 1:**
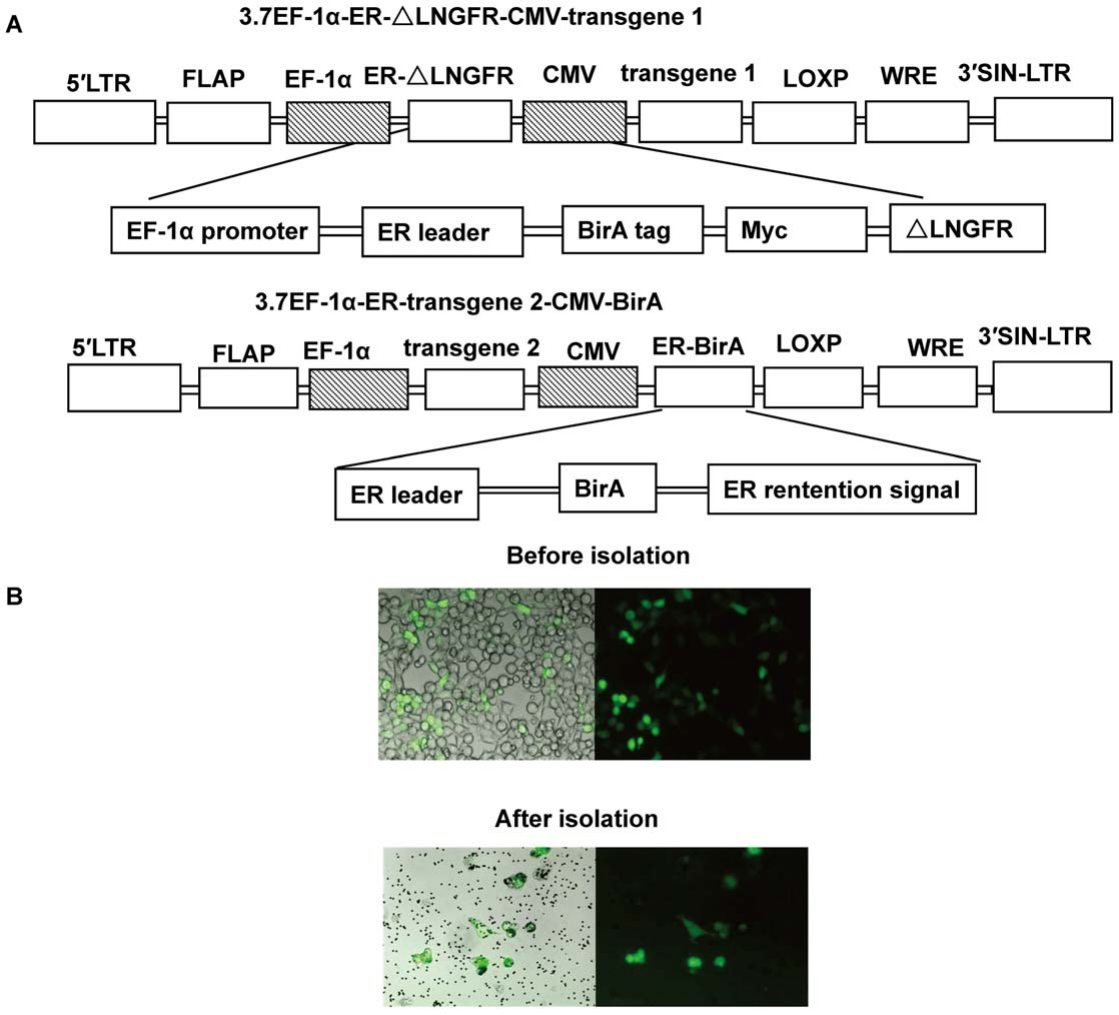
Isolation of genetically-modified cell lines with streptavidin-labeled magnetic beads. (**A**) Schematic structures of the lentivectors used for delivery of the genes of interest into cells. 3.7EF-1α-ER-**Δ**LNGFR-CMV-transgene was a lentivector derived from the backbone of pll3.7. The first transgene is expressed in a cassette with an CMV promoter. A truncated form of the membrane-anchored human low-affinity nerve growth factor receptor (LNGFR) tagged with BirA tag (GLNDIFEAQKIEWHE) peptide was driven by a EF-1α promoter in a separate cassette. 3.7EF-1α-transgene-CMV-ER-BirA was a lentivector containing the second transgene expression cassette and the BirA enzyme as a reporter gene. (**B**) The 293T cells were co-transfected with 3.7lnGFP and 3.7CMVBirA lentivectors and isolated with streptavidin-labeled magnetic beads. Merged images (left panels), GFP fluorescence images (right panels).

To express a biotinylated tag on the cell surface, we constructed a tag protein composed of an ER leader sequence and a biotin tag peptide, the biotinylation of which can be catalyzed by co-expressed BirA, and a transmembrane protein fragment with a built-in Myc tag. Initially, both the HLA-A2 transmembrane protein and a truncated form of LNGFR were tested, and LNGFR was found to express the tag protein more efficiently [23]. Therefore, a truncated form of LNGFR containing the transmembrane domain and cytoplasmic tail was used as a fusion partner for the expression of the BirA substrate at the cell surface. As depicted in Fig. 1A, two lentivectors were constructed. Vector 1 contained a CMV promoter-driven transgene expression cassette and an EF-1alpha promoter-driven truncated LNGFR tagged with the BirA substrate peptide reporter. Vector 2 contained a second transgene cassette and the BirA enzyme to catalyze the biotinylation of the BirA peptide tag at the cell surface when co-expressed with the BirA substrate peptide in Vector 1. To test the vector system, a GFP reporter gene was inserted into the transgene expression cassette of Vector 1, termed 3.7lnGFP, and a lentivector containing the BirA expression cassette without a transgene was created and named 3.7CMVBirA. Both lentiviruses were produced in 293T cells using standard procedures.

**Figure 2:**
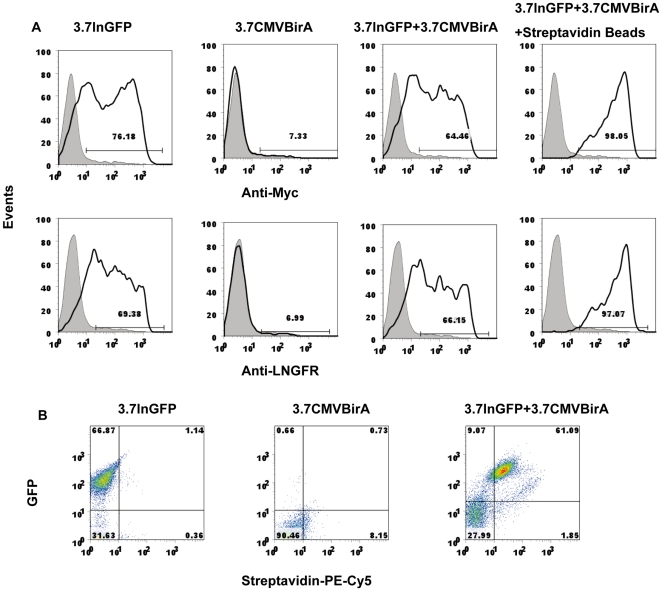
FACS analysis of genetically-modified cell lines. (**A**) FACS staining of transfected 293T cells with antibodies against the marker proteins LNGFR and Myc. 293T Cells were transfected with 3.7lnGFP, 3.7CMVBirA, or both 3.7lnGFP and 3.7CMVBirA and selected with streptavidin-labeled beads. The cells were then analyzed by flow cytometry to detect the level of surface expression of Myc or LNGFR. Histogtams indicate Myc and LNGFR expression (top four panels, middle four panals), shaded histograms represented untransfected 293T cells. The percentage of positive cells is indicated in each panel. (**B**) Flow cytometric analysis of coexpression of GFP and biotinylated ΔLNGFR. The cells were stained with streptavidin-PE-Cy5. GFP and streptavidin double positive cells are indicated by gate and percentage. Shown are the data represented as a dot-plot analysis of stained tansfected 293T cells.

To confirm that a biotinylated peptide tag was expressed **on** the cell surface, 293T cells were first transfected with the two lentiviruses, 3.7lnGFP and 3.7CMVBirA. The surface expression of biotinylated LNGFP was confirmed by binding to streptavidin-labeled beads 72 hours after transfection. As shown in Fig. 1B, cells expressing the biotinylated tag could be isolated with streptavidin-labeled beads, and almost all of the cells bound to the beads expressed GFP, this is further confirmed by FACS quantification (Fig. 2B). The surface expression of **Δ**LNGFR and Myc was also verified by FACS. As shown in Fig. 2A, cells transfected with 3.7lnGFP and cells transfected with both 3.7lnGFP and 3.7CMVBirA were stained with either anti-Myc or anti-LNGFR antibodies. The positive percentage stained with anti-Myc was 76.18% (3.7lnGFP) and 64.46% (both 3.7lnGFP and 3.7CMVBirA ) respectively, while the ones stained with anti-LNGFR was 69.38% (3.7lnGFP) and 66.15% (both 3.7lnGFP and 3.7CMVBirA) respectively. The cells transfected with 3.7CMVBirA alone were not stained by both antibodies above the background level of 7.33% and 6.99% respectively. Histogram analysis of flow cytometry data showed a broad range of staining intensity of non-isolated cells; however, the cells isolated with streptavidin-labeled beads formed a distinct population, the positive percentage being 98.05% (anti-Myc) and 97.07% (anti-LNGFR). The percentage of GFP positive cells is 68% (3.7lnGFP) or 70.16% (both 3.7lnGFP and 3.7CMVBirA) individually, and the percentage of streptavidin positive cells was 62.94%. Almost 87% GFP positive cells could be stained with streptavidin (Fig. 2B).

**Figure 3:**
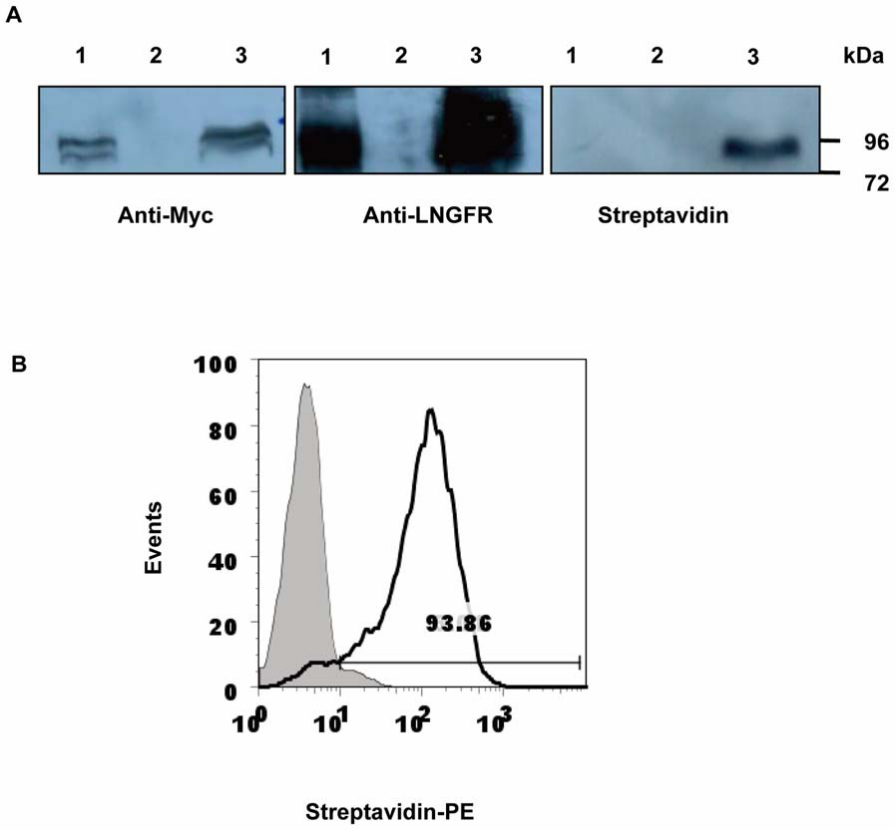
Expression and biotinylation of the transmembrane protein ER-ΔLNGFR. (**A**) Western blot analysis of the expression and biotinylation of the transmembrane protein ER-ΔLNGFR. 293T cells were transfected with 3.7lnGFP lentivector alone or in combination with 3.7CMVBirA lentivector. Forty-eight hours after transfection, the transfected cells and control untransfected 293T cells were lysed in SDS sample buffer, and the samples were separated on 10% SDS-PAGE gels and transferred to blotting membranes. The blots were probed with anti-Myc or anti-LNGFR (CD271) antibodies and streptavidin-labeled HRP and visualized by ECL. lane 1, 3.7lnGFP transfected 293T; lane 2, untransfected 293T; lane 3, 3.7lnGFP and 3.7CMVBirA cotransfected 293T. (**B**) FACS analysis of a biotinylated transmembrane protein. HCT-15 cells were infected with both 3.7lnGFP and 3.7CMVBirA lentivirus (black line ) or with 3.7lnGFP alone as a negative control (shaded histogram). The infected cells were stained with streptavidin-PE and analyzed using a flow cytometer.

In the following experiments, western blots were performed to confirm whether biotinylation occured only when both BirA and the BirA substrate peptide tag were expressed by the same cell. As shown in Fig. 3A, cells that expressed the BirA substrate peptide tag alone or in combination with BirA could be stained with either anti-Myc or anti-LNGFR antibodies. In contrast, only cells expressing both proteins were stained with streptavidin, indicating that biotinylation took place only when both proteins were expressed in the same cell. FACS analysis (Fig. 3B) strengthened the results as 93.86% cells expressed biotinylated proteins on the surface of cells.

### 2. Isolation of the gene-modified cell lines with streptavidin-labeled magnetic beads

**Figure 4:**
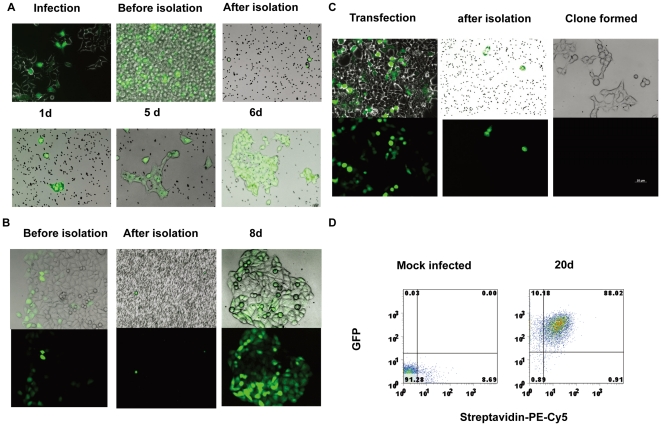
The efficient establishment of genetically-modified clones. (**A**) 293T clones expressing GFP were rapidly established by infection and sorting with magnetic beads. The 293T cells were first lentivirally transduced with 3.7lnGFP and 3.7CMVBirA. After infection, 293T cells expressing GFP were dissociated with 0.5 mM EDTA and mixed with streptavidin-labeled beads. Only GFP-positive cells adhered to the streptavidin-labeled beads. Single 293T cells isolated with the streptavidin-labeled beads were cultured to form a stable cell line within ten days. (**B**) HCT-15 clones expressing GFP were established by infection and sorted with magnetic beads. HCT-15 cells were infected with 3.7lnGFP and 3.7CMVBirA. After infection, the GFP-positive HCT-15 cells were isolated with streptavidin-labeled beads. (**C**) Establishment of a 293T cell line stably expressing BirA. 293T cells were first infected with 3.7CMVBirA lentivirus, then transiently transfected with 3.7lnGFP vector and mixed with streptavidin-labeled beads. The modified cells were isolated using streptavidin-labeled magnetic beads. (D) Stable expression of transgene in HCT-15 cells. After 20 days of selection, the stable expression of GFP was determined by FACS analysis. Shown are the data represented as a dot-plot analysis.

In the next set of experiments, the efficient isolation of clones containing transgenes was demonstrated. As shown in Fig. 4A, 293T clones that expressed GFP were quickly established by infection and sorting with magnetic beads. The 293T cells were first infected with 3.7lnGFP and 3.7CMVBirA lentiviruses. After infection, the GFP-expressing 293T cells were isolated using streptavidin-labeled beads. Only GFP-positive cells adhered to the streptavidin-labeled beads. Single 293T cells isolated with streptavidin-labeled beads were further cultured, and single cell-derived clones were established within a period of a few days. Similarly, HCT-15 clones expressing GFP were established by infection and magnetic bead sorting. After infection with 3.7lnGFP and 3.7CMVBirA, GFP-positive HCT-15 cells were isolated with streptavidin-labeled beads, and a clone was established in one week (Fig. 4B). Long term and stable expression of transgene was investigated by FACS analysis of GFP expression, the percentage of GFP-positive cells at day 20 was 98%, and 88% GFP positive cells can be stained with streptavidin (Fig. 4D).

One application for this system is the expression of biotinylated proteins in established 293T cells expressing the BirA enzyme. To establish a 293T cell line stably expressing the BirA enzyme, 293T cells were first infected with 3.7CMVBirA lentivirus. Next, the same cells were transiently transfected with 3.7lnGFP vector, and the modified cells were isolated with streptavidin-labeled magnetic beads (Fig. **4**C).

### 3. HCT-15 cells modified with a single transgene (β2m-gp100) were specifically targeted by TCR-modified NK cells

**Figure 5:**
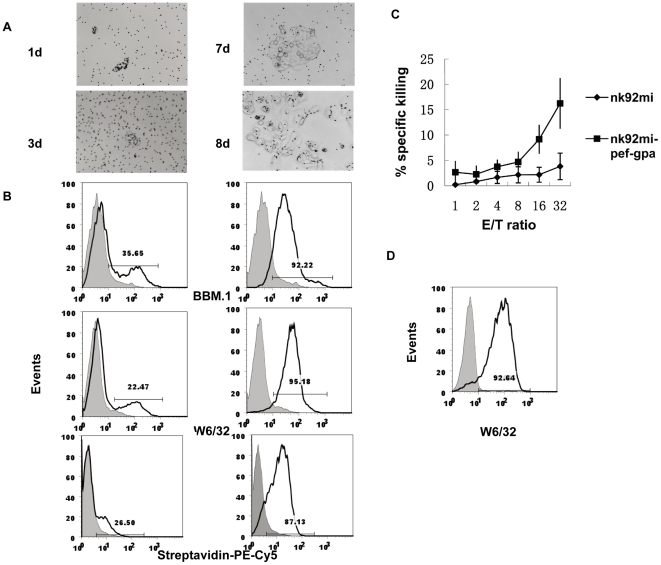
HCT-15 cells modified with a single transgene (β2m-gp100) were specifically targeted by TCR-modified NK cells. (**A**) Modified HCT-15 cells were isolated with streptavidin-labeled beads, and single cell-derived clones were grown for 8 days. (**B**) FACS analysis of HCT-15 cells transduced with 3.7ln β2mgp100 and 3.7cmvBirA lentivirus. The cells were stained with mAbs W6/32, which is specific for HLA-A, B and C, BBM1, which is specific for β2m and streptavidin-PE-Cy5 conjugate. Mock-infected HCT-15 cells were infected with lentivirus without a target gene (shaded histograms). HCT-15 cells were infected with lentivirus 3.7ln β2mgp100 and 3.7CMVBirA (left panels) and selected by streptavidin-labeled beads (right panels). (**C**) Specific killing of HCT-15 cells expressing β2mgp100 by NK92MI/gp100 cells. NK92MI-pef-GPA7A is a NK92MI cell line expressing GPA7A, a TCR-like antibody specific for HLA-A2 loaded with gp100 peptide. The parental NK92MI cell line was used as a control. The results are presented as percentage ±SD from five individual experiments. (**D**) Stable expression of β2mgp100 gene in lentivirus transduced HCT-15 cells. Transduced cells were passed 20 passages and stained with W6/32 antibody (black line). Mock-transduced HCT-15 served as a negative control (shaded histogram).

To further demonstrate the efficiency of transgenic cell isolation, we transduced HCT-15 cells with β2m linked to a peptide derived from gp100 (β2m-gp100). HCT-15 cells are β2m-deficient human colorectal cancer cell line. Because they lack β2m, no stable HLA I molecules are expressed at the cell surface. As shown in Fig. **5**B, non-transduced HCT-15 cells did not express HLA class I molecules at the surface. HCT-15 cells were infected with two lentiviral vectors: one expressing β2m-gp100 and the BirA substrate peptide tag and another expressing the BirA enzyme. Biotinylated cells were isolated with magnetic streptavidin-labeled beads, and clones were established (Fig. 5A). The presence of β2m on the surface of HCT-15 cells was confirmed by FACS analysis using W6/32 or BBM.1 antibodies, wherein approximately 92.22% (BBM.1) and 95.18% (W6/32) of transduced cells were stained (Fig. 5B, top and middle panels). Biotinylated proteins at the cell surface were detected with streptavidin-PE-Cy5, and observed staining in approximately 87.13% of transduced cells (Fig. 5B, bottom panels). We further tested the stability of β2m/gp100 expression in HCT-15 cells using FACS analysis. 20^th^ passage of established β2m/gp100-positive HCT-15 cells were stained with mAb W6/32 ( Fig. 5D) of which 92.64% were positive.

The established β2m/gp100-positive HCT-15 cells were further tested as target cells for NK92MI cells expressing GPA7A, a T cell receptor (TCR)-like antibody specific for a gp100 peptide-loaded HLA-A2 complex. As shown in Fig. 5C, the established β2m/gp100-positive HCT-15 cells were effectively targeted by GPA7A-transfected NK92MI cells, which specifically recognized target cells expressing the gp100 peptide-loaded HLA-A2 complex. In contrast, parental NK92MI cells induced a much lower level of cytotoxicity.

### 4. The establishment of cell lines expressing two sets of desired transgenes

**Figure 6:**
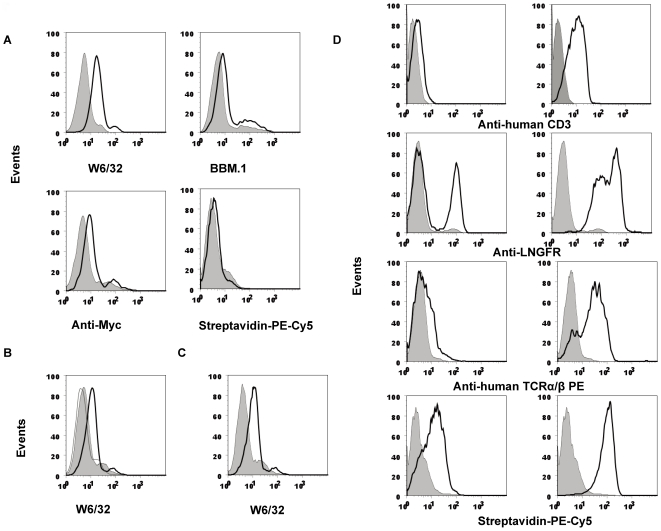
Efficient establishment of double transgenic cell lines. (**A**) HLA-A2 and β2m gene expression in the K42-91a cell line. K42-91a cells transduced with HLA-A2 and β2m were stained for transgene expression with anti-Myc antibody and streptavidin-PE-Cy5, and the expression of HLA-I molecules was determined using the mAbs W6/32 and BBM1. Mock-transduced K42-91a served as a negative control (shaded histogram). (**B**) Cell lines expressing both HLA-A2 and β2m (shaded histogram) were loaded with an HLA-A2-restricted peptide epitope derived from the melanoma protein Mart1 (black line) using an HLA-B27 peptide as a control (grey line). (**C**) Stable expression of HLA-A2 and β2m in K42-91a cells. Transduced cells were passed 15 passages and stained with W6/32 antibody (black line). Mock-transduced K42-91a served as a negative control (shaded histogram). (**D**) Establishment of T cell lines expressing TCR genes. TCRα and TCRβ genes specific for the gp100 peptide were transduced into SupT1 cells using two lentiviruses. Transduced SupT1 cells were isolated with streptavidin-labeled beads. The established SupT1 cells were stained with anti-human TCRα/β, anti-human CD3, and anti-LNGFR antibodies or streptavidin-PE-Cy5 conjugate. Left panels: before isolation, right panels: after isolation. Mock transduced-SupT1 cells served as a negative control (shaded histogram).

The potential of this system was further realized by the efficient establishment of cells modified with two sets of transgenes. The individual genes were cloned into separate lentiviral vectors and introduced into target cells, and stably transduced cell lines were then isolated. Two pairs of genes, a combination of human β2m and HLA-A2 and the combination of melanoma associated-Ag gp100-specific TCR-alpha and -beta chains, were used to establish **the** cell lines. The K42-91a cell line [24], a variant of the K42 mouse fibroblast cell line in which TAP2 has been knocked down with a targeted knockout of the CRT gene, was used to establish gene-modified cells expressing the HLA class I complex. Lentiviruses expressing HLA-A2 and human β2m were used to infect K42-91a cells, the cells modified with both HLA-A2 and β2m were isolated using magnetic streptavidin-labeled beads, and stable clones were established. Staining with anti-HLA complex antibodies showed that human HLA I complexes were expressed on the surface of murine k42-91a cells (Fig. 6A, top two panels), the cells were also stained with anti-Myc antibody and streptavidin-PE-cy5 (Fig. 6A, bottom two panels). The surface expression of the human HLA I complex on k42-91a cells was further confirmed by flow cytometry, which showed a shift to the right in the human HLA I staining profile. In contrast, the staining profile of an irrelevant peptide did not change (Fig. 6B). To confirm stable expression of HLA-A2 and human β2m in K42-91a cells, 15^th^ passage of established HLA-A2 and β2m transduced K42-91a cells were analyzed using FACS ( Fig. 6C).

In another set of experiments, SupT1 cells were modified to express TCR genes. Wild-type SupT1 cells are TCR-deficient, and although all of the genes for the CD3 components are present, these cells do not express surface CD3 because of the lack of the TCR. The TCR is composed of alpha and beta chains and is MHC restricted. The CD3 complex is expressed on the surface of SupT1 cell when both of the TCR subunits are re-expressed. The TCR-alpha and -beta chains derived from melanoma-specific T cells were inserted into the transgene expression cassettes of the respective lentivectors, and viruses expressing the transgenes were produced [25]. SupT1 cells were infected with both a virus containing the TCR-alpha gene and a virus containing the TCR-beta gene. After isolation with magnetic streptavidin-labeled beads, the cells were further cultured, and the expression of the modified genes was confirmed by FACS analysis. As presented in Fig. 6D, CD3, LNGFR, TCR-alpha and -beta chains and biotinylated transmembrane proteins were clearly expressed on SupT1 cells due to the integration of both the TCR-alpha and -beta genes mediated by the lentiviral vectors. Homogenous SupT1 cells expressing TCR were obtained after infection (Fig. 6D, left panels) and magnetic bead separation (Fig. 6D, right panels).

## Discussion

The development of cell-based gene therapy and the production of recombinant proteins for clinical applications both require genetically modified cells. These applications are largely hampered by the lack of protocols for the simple isolation of genetically modified cells. In this report, we have described a new method that permits the specific and efficient isolation of gene-modified cells based on the strong and specific interaction between biotin and avidin [13]. In our system, mammalian cells are modified with two separate vectors, one expressing the BirA enzyme, which has been engineered to be retained in the endoplasmic reticulum (ER), and the other expressing as a transmembrane protein with a BirA substrate peptide tag [26], [27], [28], [29], [30], [31], [32], [33], [34]. The BirA substrate peptide tag will be expressed on the surface of a cell when both genes are expressed along with two sets of target genes. The resulting biotin-labeled cells can be isolated using magnetic streptavidin-labeled beads. In this way, genetically modified cells can be obtained as early as 72 hours post-infection. The viability of the cell lines is not affected, and there is no toxic effect on cell proliferation [35], [36], [37].

Gene therapy depends on both efficient gene transfer into the appropriate target cells and the ability to enrich the transduced cells. Conventional selectable markers have limitations, including growth inhibition, mutations in antibiotic resistance genes, cell membrane permeability and morphological changes. **Δ**LNGFR is a truncated native receptor that is not immunogenic, does not mediate signal transduction and is already being used in clinical protocols [38]. **Δ**LNGFR is easily tracked in vivo, and cells expressing this transgene can be isolated using magnetic or fluorescence-activated cell sorting. The BirA enzyme is a foreign protein introduced into the cells and is designed as an ER-resident protein [22]. Although no cytotoxic effects have been reported upon introduction of BirA into cells, its potential effects on cell cycle and its long-term safety require further investigation [28].

The human immunodeficiency virus type-1(HIV-1) based lentiviral vectors provide high and stable transgene in both dividing and non-dividing cell types, the third generation self-inactivating (SIN) vectors minimize the risk of reconstituting a replication-competent lentivirus (RCL). In our lentiviral system, two ubiquitously internal promoters in lentivectors, human elongation factor-1α (EF-1α) promoter [39], [40] and cytomegalovirus (CMV) promoter [35], [41], were chosen to drive transgene expression, and stable cell lines were generated in different cell types. We also tested whether transgene expression could be stably maintained when transduced cells are expanded where we observed that transgenes were stable expressed after at least 15 passages in different cell types. However, silencing of vector transgene is an important factor to consider where long term stable expression of transgene is required in target cells. There are specific reports stating many transgenes driven by viral promoter including the CMV promoter have been found gradually silenced over period of months in certain cell types [42]. Replacement of CMV promoter by appropriate cell-type specific promoters when needed facilitate efficient transgene manipulation and isolation of gene-transducted cells.

Our results show that the use of this selective system allows **for** rapid, efficient, and nontoxic ***in vitro*** selection of gene-modified cell. This system has been applied in many different cell lines and will provide an option in isolating tranfected cells, and set to use the system to select the TCR-alpha and -beta genes derived from melanoma-specific T cells transduced human primary T cells which has future prospects to treat melanoma. More importantly, the selective system can be used to design vaccine, owing to gene-modified DC vaccine expressing TAA, gene encoding cytotoxic T lymphocyte (CTL)-specific epitopes, and are promising vaccine candidates to be utilized for treatment of cancer [43], [44], [45], In summary, this methodology provides many potential of application in adoptive gene therapy, producing recombinant protein and studying the function of genes.

## Materials and Methods

### Lentiviral Vector construct

We used the backbone of the pLL3.7 plasmid (Invitrogen, Carlsbad, CA, USA) (Fig. 1A) to construct the 3.7transgene 1BirA and 3.7lntransgene 2 lentivectors, termed 3.7BirA and 3.7ln, respectively (Fig. 1). To obtained EF-1α-ER-**Δ**LNGFR fragment, the ΔLNGFR sequence was amplified from the template pPRIME-CMV-LNGFR-cloning vector( PMID 16141338) using primer: LN-*Pst*I, 5′ GCATGGACTGCAGGAACAAAAACTCATCTCAGAAGAGGA 3′; LN-1,5′ CAT CTCAGAAGAGGATCTGGGA GGTGCATGCCCCACAGGCCT 3′ and LN-*Not*I, 5′ GATAAGAATGCGGCCGCTCTAGAACTAGT 3′, was cloned into PEF/Myc/ER vector (Invitrogen, USA), and then the EF-1α-ER-**Δ**LNGFR was inserted into *Xba*I and *Not*I of pll3.7 plasmid to get 3.7EF-1α-ER-**Δ**LNGFR-CMV-GFP (3.7lnGFP) which u6 promoter–driven siRNAs expressing cassette was replaced. GFP gene can be replaced with foreign transgenes. The BirA enzyme gene was amplified by PCR from the POZ-BirA plasmid(previously constructed in our lab), The primers used to amplify BirA were BirA-*Sal*I, ACGCGTCGACATGAAGGATAACACCGTGCC ACTGAA and BirA-*Not*I, AAGGAAAAAAGCGGCCGCGTTTTTTGCACTAC GCAGGG. The amplified fragment was ligated into the *Sal*I and *Not*I sites of the PEF/Myc/ER vector. The stop codon of BirA was deleted to introduce an ER retention signal at the C terminus of BirA, termed ER-birA. Next, the ER-BirA gene was subcloned by digesting PEF/myc/ER-BirA with *Nhe*I and *EcoR*I and inserting the fragment containing ER-BirA into the pll3.7 lentiviral vector. For expression of a single transgene, the GFP or β2m-gp100 (a gp100 epitope-linked hβ2m sequence ) gene was cloned into the 3.7ln vector at the *Nhe*I and *EcoR*I sites. For expression of two transgenes, the TCR-alpha and -beta chain genes, HLA-A2, as well as the human β2m genes were cloned into the 3.7ln and 3.7BirA vectors respectively. The *Sph*I and *BamH*I sites were used for cloning into the 3.7BirA vector. The primers used to amplify gp100-TCR from APB plasmid (a gift from Dr. Richard Morgan) were as follows: TCR-A-*Pml*I(+), 5′ GTCACG TGGCCA CCATGGTGAAGATCCGGCAA 3′; TCR-A-*BamH*I(−), 5′ CGCGGATCCTCAGCTGGACCACAGCCGCA3′; TCR-B-NheI(+), 5′CTAGCTAGCCACCATGGACT CCTGGACCTTC TGCTG3′; and TCR –B –*EcoR*I (−) 5′CCGGAATTCTCATCCCCTTCTTCTGAGAC3′. The primers β2m-*Nhe*I-h, 5′ATCGGCTAGCATGTCTCGCTCCGTGGCCTT3′ and β2m -*Mun*I-h, 5′ ATGCA TCAATTGTCACATGTCTCGATCCC 3′ were used to amplify human β2m. We then used the primers, A2-*Sph*I, 5′ ATGCATGCGCCACCATGGCCGTCAT GGC G 3′ and A2-*BamH*I, 5′ CGCGGATCCTCACACTTTACAAGCTGTGAGAG 3′ to generate the HLA-A 2 chains. Both hβ2m and HLA-A 2 genes were amplified from pCDNA3.1A2B2 plasmid (previously constructed in our lab).

### Cell lines and antibodies

The HEK293T cells and the HCT-15 colon adenocarcinoma cells used in this study were cultured in Dulbecco's modified Eagle medium (DMEM) supplemented with 10% fetal bovine serum (Gibco BRL, Caithersburg, MD, USA). The T lymphoid SupT1, NK92MI and K42-91a cell lines were cultured in RPMI-1640 medium supplemented with 10% fetal bovine serum (FBS) and 100 U/mL penicillin/streptomycin. All cell lines were purchased from ATCC (Rockville, MD, USA) except HCT-15 cells (a gift from Chen Au Phe) and K42-91a cell line [24]. All transformants were supplemented with biotin at a final concentration of 100 µM. Cells were maintained in 5% CO_2_ in a 37°C incubator. W6/32, BBM.1, anti-Myc, mouse anti-Human CD271 (anti-LNGFR), anti-human-CD3-FITC and anti-human-TCR-α/β-PE antibodies were obtained from MACS (Miltenyi Biotec, Cologne, Germany). Anti-mouse-IgG-PE-Cy5, HRP conjugated goat anti-mouse IgG and anti-mouse-IgG-FITC antibodies were obtained from Sigma-Aldrich (St Louis, Mo, USA). Streptavidin-PE-Cy5 conjugate and streptavidin-PE conjugate were purchased from eBiosicence (San Diego, CA, USA). HRP-conjugated streptavidin was from BD PharMingen (San Diego, CA, USA).

### Production and concentration of lentiviral vectors, transduction and selection of transfected cells

VSV-G pseudotyped lentiviral vectors stocks were prepared, concentrated, and titered as described previously [46]. Briefly, 293T cells were transiently transfected with 6 µg of packaging plasmids pLP1, pLP2 and pLP/VSVG (Invitrogen, Carlsbad, CA, USA) respectively and 6 µg of transfer vector. After 48 hours, virus-containing supernatants were harvested, passed through a 0.45 µm filter and frozen in aliquots at −80°C until used. The virus was used to transduce different cell lines in the presence of 4 µg/mL polybrene at different the multiplicity of infection (MOI). Adherent cells (293T, HCT-15, K42-91a) were transduced at an MOI of 1, suspension cells (supT1) were transduced at an MOI of 20. All cells were transduced by spin inoculation in a centrifuge at 1,800× g for 90 minutes at 32°C in 6-well tissue culture plates (2 mL per well) and incubated at 37°C overnight; fresh medium was added after the overnight incubation. This process was repeated once after every 24 hours later. A mock infection (no vector-containing medium) was always performed in parallel under identical culture conditions. Magnetic bead-based cell separation was performed 72 hours post-infection. After the lentiviral infection and separation of transduced cell lines, the transduction efficiency was monitored by flow cytometry.

### Magnetic bead-based cell separation

To avoid potential cleavage of surface proteins by trypsin, the adherent cells were released from the plates with 0.5 mM EDTA. The cell suspension **was** washed 3 times to remove unbound d-biotin. Next, Dynabead M-280 streptavidin-labeled beads (Invitrogen, Carlsbad, CA,USA) were mixed with the cell suspension at a ratio of 10 µl bead suspension per 1 mL (1×10^7^) target cell suspension. The resulting mixture was rotated for 20 minutes on ice. The tubes were placed into a magnetic device for 5 minutes, and the bound cells were collected. Tubes were removed from the magnet, and the beads were washed once with phosphate-buffered saline (PBS) / 2% FBS. The collected cells were harvested and resuspended in 1.5 mL PBS (pH 7.4). These procedures were repeated three or four times. The biotin-positive cells were separated from the suspension with a magnetic unit. The presence of the biotinylated surface marker on the harvested cells was confirmed either by additional staining of the cells with antibody or by observation of GFP in unstained cells under a fluorescence microscope.

### SDS-PAGE and western blotting

One million cells were lysed and centrifuged, and the supernatants were boiled in SDS sample buffer for 5 minutes and separated by SDS-PAGE in a 12% polyacrylamide gel. The proteins in the gel were then transferred to Hybond C Extra membrane (Amersham) at 20 V for 2 hours. The membrane was blocked in 10 mL of PBS containing 2% Marvel milk (Premier International Foods, St Albans, UK) on a plate rocker (Stuart Gyro Rocker STR-9) for 1 hour and transferred to 10 mL of Phosphate Buffered Saline with Tween 20 (PBST) /2% Marvel milk containing a 1∶1500 dilution of the primary antibody. The membrane was washed 3 times and transferred to PBST/2% Marvel milk containing a 1∶5000 dilution of the HRP-conjugated secondary antibody. The membrane was washed twice in PBST and twice in PBS and dabbed dry with a paper towel. Two milliliters of enhanced chemiluminescence fluid were added evenly to the membrane. After 5 minutes, the fluid was pipetted off, and the membrane was dabbed dry which was then wrapped in Parafilm and attached to one side of an exposure box with tape. Photographic film (Kodak Biomax) was exposed to the membrane for 10 seconds to 30 minutes.

### Flow cytometry analysis

W6.32, BBM.1, anti-LNGFR and anti-Myc were used as primary antibodies. The cells were washed 3 times with PBS, and 1×10^6^ cells were incubated with mAbs on ice for 30 minutes and then incubated with or without secondary antibody (mouse IgG conjugated to FITC or PE-Cy5). The anti-human-TCR-α/β-PE antibody was used according to the manufacturer's instructions. After each incubation, the cells were washed twice with PBS. The labeled cells were analyzed by flow cytometry with a Guava Easycyte (Guava Technologies, Hayward, CA, USA). All FACS data were analyzed using FlowJo software (Tree Star, Ashland, OR, USA).

### Cytotoxicity assay

Target cells were washed twice with Hank's buffered salt solution (HBSS) before staining. The 1–2×10^7^ cells were then incubated for 10 min with Hank's buffered salt solution (HBSS) supplemented with 2 µM carboxyfluorescein succinimidyl ester (CFSE, Invitrogen, Merelbeke, Belgium) at 37°C in the dark. Immediately following the addition of the CSFE, an equal volume of FBS was added to stop the reaction. After incubation with FBS for 1 min, the target cells were washed twice with HBSS and resuspended in complete medium at a concentration of 1×10^5^ cells /mL; 100 µl of each sample was added to each well of a 96-well U-bottom plate. Triplicate samples containing mixtures of effector and target cells at E∶T ratios of 1∶1, 2∶1, 4∶1, 8∶1, 16∶1, and 32∶1 were prepared. The cells were incubated at 37°C for 4 hours in the presence of 5% CO_2_. After incubation, the cells were transferred to a 1.5 mL tube, centrifuged at 250× g for 5 minutes, washed once with ice-cold medium and resuspended in 500 µl ice-cold medium. The cells were incubated for 3 minutes on ice with 10 µg/mL propidium iodide ( PI; Sigma-Aldrich, Poole, England) and both green and red fluorescence were analyzed. Cells stained with both dyes represent dead target cells. Analysis was on gated CFSE positive cells only. The percentage of cytotoxic activity was calculated using the following formula: %specific death = (%total CFS^+^PI^+^ (dead) cells−%spontaneous CFSE^+^PI^+^ (dead) cells)/(100%−%spontaneous CFSE^+^PI^+^ (dead)cells)×100%. The percentage of spontaneous dead cells was measured in the tube containing target cells only [47].

### Image Acquisition

Images were acquired on a ZEISS Axiovert 200 M microscope (Zeiss, Vienna, A) with a 40× NA 0.55 objective. The microscope and image acquisition were controlled by Axiovision software. For GFP observation we used the blue light excitation. Image processing, such as clipping of images, image montages etc. was performed in PowerPoint.
